# Aflatoxin decontamination in tree nuts: a scoping review of physical, chemical, and biological methods

**DOI:** 10.1007/s12550-026-00664-y

**Published:** 2026-08-04

**Authors:** Hanna Lemos, Cinthia de Carvalho Couto, Fábio de Oliveira Borges, Ariane Kluczkovski, Otniel Freitas-Silva

**Affiliations:** 1 Federal Rural University of Rio de Janeiro, Seropédica, Brazil; 2 UNIRIO – Graduate Program in Food and Nutrition (PPGAN/UNIRIO), Rio de Janeiro, Brazil; 3 Institute of Physics, Fluminense Federal University, Niterói, Brazil; 4 Federal University of Amazonas, Manaus, Brazil; 5 Embrapa Agroindústria de Alimentos, Rio de Janeiro, Brazil

**Keywords:** Aflatoxins, Detoxification, Decontamination, Nuts

## Abstract

Aflatoxin (AF) contamination in tree nuts poses a serious threat to global food safety, public health, and international trade due to the potent carcinogenicity of aflatoxin B_1_ (AFB_1_). Although traditional mitigation strategies exist, their industrial implementation is constrained by a strict “technological filter,” in which high detoxification efficacy must be carefully balanced against the preservation of the nutritional, structural, and sensory quality of the food matrix. This scoping review systematically mapped and critically synthesized recent scientific advances (2005–2025) in physical, chemical, and biological decontamination methods, evaluating their operational effectiveness, underlying mechanisms, and qualitative impacts on tree nuts. Guided by the question, “What physical, chemical, and biological methods are most effective for AF decontamination in tree nuts, and to what extent are they feasible regarding quality preservation and industrial applicability?”, the study strictly followed the Joanna Briggs Institute (JBI) Manual for Evidence Synthesis and was reported according to PRISMA-ScR guidelines. A total of 41 eligible original studies were selected after comprehensive screening of the PubMed, Scopus, Web of Science, and ScienceDirect databases. Physical approaches, particularly cold atmospheric plasma and UV-C radiation, achieved robust reduction rates ranging from 70% to 95%; however, these highly energetic treatments often induced lipid oxidation, evidenced by linear increases in malondialdehyde (MDA) levels in matrices such as pistachios. Among chemical methods, ozonation and the use of organic acids achieved degradation rates close to 100% for the most toxic forms, but significant technological trade-offs were identified, including up to a 29% loss of α-tocopherol in hazelnuts. Inorganic selenium emerged as a promising alternative by combining efficacy with matrix preservation. Biological strategies using microorganisms such as *Bacillus subtilis*, *Bifidobacterium lactis*, and *Lactobacillus kefiri* demonstrated substantial sustainable potential, with detoxification efficiencies exceeding 80% through dual mechanisms of active biosuppression and physical adsorption. No single universal method is currently sufficient to handle contamination safely. The future of commercial AF management depends on the development of “hurdle technology,” integrating multi-stage synergistic interventions with automated optical sorting and intelligent packaging, supported by further technical advances, studies of practical applicability, and industrial-scale validation to ensure absolute consumer safety and commercial viability.

**Graphical Abstract:**

**Figure:**
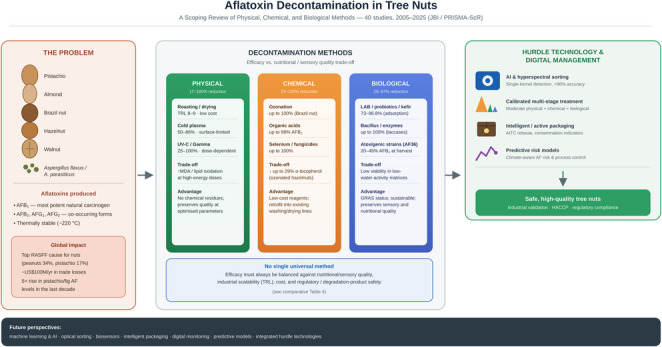

## Introduction

Tree nuts are highly susceptible to infection by fungi of the genus *Aspergillus*, particularly the species *Aspergillus flavus* and *Aspergillus parasiticus*. Such contamination can occur ubiquitously at all stages of the production chain, including harvesting, storage, and processing (Ma et al. 2021). *Aspergillus* fungi survive in agricultural soils and infect various crops, producing aflatoxins (AFs) that contaminate not only solid products but also water resources (He et al. 2023). Climatic conditions characterized by high humidity and temperatures exceeding 30 °C, common in producing regions and exacerbated by global warming, create the ideal microenvironment for spore germination. Such climate changes is projected to intensify this risk further, rising mean temperatures and increasingly erratic rainfall patterns are expected to expand the geographical range suitable for *A. flavus* proliferation and AF biosynthesis into historically temperate nut-producing regions, while also altering pre-harvest drought-stress patterns that are themselves associated with higher aflatoxin accumulation, compounding the challenge of post-harvest decontamination (Kluczkovski et al. 2025). The risk is maximized when the nuts remain in prolonged contact with moist soil during harvest, allowing fungi to penetrate the nut matrix and initiate accelerated toxin biosynthesis even before industrial processing begins (Costa et al. 2017; Kluczkovski et al. 2025).

AFs are toxic, carcinogenic secondary metabolites with high chemical stability that induce tumor development by forming DNA adducts with guanine (Wang et al. 2023). More than 20 types of AFs have been identified, with AFB_1_, AFB_2_, AFG_1_, and AFG_2_ being the most prevalent, and AFB_1_ is classified as the most potent natural carcinogen, exhibiting toxicity up to 68 times greater than that of arsenic (Kępka-Borkowska et al. 2025). Beyond the direct ingestion of contaminated seeds and grains, these toxins can be metabolized by animals and transferred to products such as milk (in the form of AFM_1_), thereby increasing the risk of population exposure (Goda et al. 2025). The toxicological effects are severe: acute exposure can lead to liver failure and death, while chronic exposure is linked to hepatocellular carcinoma, immunosuppression, stunted childhood growth, and neurological damage. Mycotoxin-related diseases are estimated to account for 40% of disability-adjusted life years (DALYs) lost in certain regions, with global warming exacerbating fungal proliferation in previously temperate zones (Yohannis et al. 2025).

The global economic impact of AFs is devastating, with annual losses in the nut and dried fruit industry potentially reaching $100 million due to border rejections, batch destruction, and analysis costs (Leite et al. 2023; Goda et al. 2025; Yohannis et al. 2025). Historically, an FAO estimate cited that 25% of global crops were contaminated; however, recent studies indicate that this figure is a severe underestimate, suggesting that up to 60–80% of crops may contain detectable levels of mycotoxins (Eskola et al. 2020).

Plant-based products contaminated with mycotoxins generate more notifications in the European Rapid Alert System for Food and Feed (RASFF) than other hazards. AFs, the primary fungal toxins, are most frequently associated with notifications concerning nuts and nut products (Kowalska and Manning 2021). RASFF notifications between 2011 and 2021 for “Nuts, Nut Products, and Seeds” reported 52% (*n* = 1,545) for peanuts, 27% (*n* = 795) for pistachios, 10% (*n* = 311) for hazelnuts, 5% (*n* = 149) for almonds, 0.7% (*n* = 22) for chestnuts, 0.1% (*n* = 4) for walnuts, and 0.03% (*n* = 1) for pine nuts (Owolabi et al. 2023). Notifications between 2010 and 2023 confirm that peanuts (34.4%), pistachios (17.3%), and Figs. (12.5%) pose the highest contamination risks. Notably, a fivefold increase in median AF levels was reported in pistachios from Iran and figs from Turkey over the last decade, highlighting failures in traditional control measures and the need for rigorous monitoring (Kow et al. 2025).

AFs are difficult to eliminate because of their thermal stability and decompose only at temperatures near 220 °C (Bashir et al. 2022). Although strong oxidizing agents (chlorine, ozone) and ultraviolet light can reduce their concentration, these conventional methods often alter the sensory properties or nutritional value of foods (Freitas-Silva et al. 2013). To ensure food safety without compromising technological quality, mitigation strategies have evolved toward more sophisticated physical, chemical, and biological methods. Their efficacy depends on factors such as the chemical stability of the mycotoxins, the nature of the process, the type of food matrix and its interactions, and interactions involving multiple mycotoxins (Rushing and Selim 2019). These methods should inactivate, destroy, or remove the toxin; avoid producing or leaving toxic residues; preserve the food’s nutritional value; not alter its acceptability or technological properties; and, if possible, destroy fungal spores to prevent proliferation and the production of new mycotoxins (Mateus et al. 2021).

Beyond conventional decontamination, food safety management is increasingly moving toward digital, technology-driven risk control. Artificial intelligence (AI) and machine learning (ML) algorithms are being incorporated into predictive models for pre- and post-harvest aflatoxin contamination risk and into rapid, non-destructive detection pipelines, with the best-performing algorithms achieving classification accuracies above 90% in several commodity matrices (Focker et al. 2025; Deshmukh et al. 2025). In parallel, hyperspectral imaging (HSI) combined with deep-learning classifiers, such as three-dimensional Inception–ResNet architectures, has demonstrated the ability to flag aflatoxin B_1_ contaminated kernels at the single-nut level in almonds, providing a non-destructive basis for automated optical sorting that could be integrated as a pre-treatment or complementary step within tree nut processing lines (Kabir et al. 2025). These “intelligent food safety” tools do not replace physical, chemical, or biological decontamination; rather, they have the potential to precede and complement them within an integrated, hurdle-technology framework, by removing the most heavily contaminated kernels before downstream treatment and by supporting real-time process control.

Despite these advances, the literature lacks a critical synthesis comparing the industrial feasibility and cost-effectiveness of these new technologies in the specific context of tree nuts. Recent reviews have addressed aflatoxin and mycotoxin decontamination in related but distinct contexts, such as nanomaterial-based detection and removal strategies across broad food and feed matrices (Ganesan et al. 2024), atmospheric cold plasma decontamination spanning grains and nuts as a single, undifferentiated category (Javed et al. 2025), decontamination specifically in corn and corn-based products (Mir et al. 2025), physical methods for reduction of aflatoxins exposure in groundnuts in low-income countries (Mshanga et al. 2023), and pre-harvest genetic resistance mechanisms in peanuts (Yang et al. 2025). However, tree nuts constitute a structurally and economically distinct commodity group, their shell architecture ranges from the highly porous, naturally dehiscent pistachio shell to the dense, sealed Brazil nut pericarp; their high lipid content makes them disproportionately vulnerable to oxidative degradation during physical and chemical treatments; and, as the RASFF data above indicate, they are among the food groups most frequently rejected at international borders because of AF contamination. No existing review has systematically mapped and critically compared physical, chemical, and biological decontamination methods specifically within this commodity group while jointly weighing detoxification efficacy against nutritional, sensory, and industrial feasibility criteria, the knowledge gap this scoping review was designed to address. Therefore, to track and compile the methods used for AF decontamination in tree nuts over the last 20 years (2005–2025), this scoping review was conducted to identify research gaps, assess the efficacy of emerging innovations, including AI-assisted detection and automated optical sorting, and propose guidelines for integrated management strategies that safeguard public health and the sector’s economic stability.

## Materials and methods

### Protocol

This scoping review was conducted in accordance with the JBI Manual for Evidence Synthesis and reported following the Preferred Reporting Items for Systematic Reviews and Meta-Analyses extension for Scoping Reviews (PRISMA-ScR) (Almeida et al. 2023; Peters et al. 2020; Tricco et al. 2018). The review was guided by the main research question: “What physical, chemical, and biological methods are most effective for AF decontamination in tree nuts, and to what extent are they feasible in terms of quality preservation and industrial applicability?”

### Eligibility criteria

Articles investigating methods for decontamination or reduction of AFs in nuts were included if published between 2005 and 2025. Studies conducted in any geographical context were eligible for inclusion. Non-original works, such as commentaries, reviews, letters to the editor, case reports, theses, and abstracts, as well as review articles, were excluded. Studies conducted exclusively on other food matrices, involving different mycotoxins, in vitro studies, and those that did not perform a before-and-after comparison of the applied methodology were also excluded. It should be clarified that purely in vitro studies conducted in synthetic culture media or chemical solutions without interaction with food were excluded to maintain a focus on the actual food matrix. However, laboratory studies using artificially inoculated nuts to simulate industrial processing conditions were retained.

### Search strategy

A search for original articles was conducted using the following electronic bibliographic databases: PubMed, Web of Science, Scopus, and ScienceDirect. Keywords were selected from the Descriptors in Health Sciences (DeHS), using the search strategy: aflatoxins AND (decontamination OR reduction) AND (nuts OR “tree nuts”), with a publication date filter set from 2005 to 2025. The search was performed in article titles, keywords, and abstracts.

The search strategy was not restricted by language. The last search was conducted on February 2, 2026. An inherent limitation of this review is that the search was restricted to the title, abstract, and keyword fields, which may have omitted relevant studies not indexed by these specific terms.

### Study selection

Study selection rigorously followed the PRISMA-ScR protocol. Two independent reviewers conducted the initial screening using Rayyan^®^ software, ensuring transparency and eliminating duplicates. The authors conducted the study selection in three phases: title screening, abstract review, and full-text reading and comprehension. Divergent decisions were resolved by consensus when necessary.

### Data extraction

From the eligible studies, the reviewers extracted relevant information by reading the full articles and creating an extraction table based on criteria established by the researchers: (i) first author’s name and year of publication; (ii) tree nuts; (iii) contamination type; (iv) processing conditions; (v) mechanisms; (vi) quality impact; (vii) applied technique; (viii) aflatoxins and (ix) efficiency /reduction.. The extracted information is presented in Tables 1, 2 and 3.

**Table 1:** Summary of the included studies applying physical methods on aflatoxin degradation

References	Tree nuts	Contamination Type	Processing Conditions	Mechanism	Quality Impact	Applied technique	Aflatoxin	Efficiency/Reduction
**Thermal**								
Yazdanpanah et al. 2005	Pistachio	Artificial	150 °C/90 min	Thermal degradation of aflatoxin molecules	Burnt appearance and adverse sensory change	Industrial roasting	AFB_1_/AFB_2_	95%
García-Cela et al. 2013	Pistachio	Natural	135 °C (pre)/165 °C (roast)/20 min	Fungal inactivation and degradation by dry heat	Compliance with legal limits without loss of commercial value	Industrial roasting	AFT	86.74%
Costa et al. 2017	Brazil nut	Natural	45 °C/6 h	Reduction of water activity and fungal inhibition	No change in physicochemical composition (except ash)	Natural convection drying	AFT	86%
Rastegar et al. 2017	Pistachio	Natural	120 °C/60 min	Heat-catalyzed acid hydrolysis of the lactone ring	Better sensory acceptance than purely thermal treatments	Roasting + citric acid/lemon	AFB_1_	93.10%
Siciliano et al. 2017	Hazelnut	Natural	140 °C/40 min	Radiative heat transfer and thermal degradation	Preservation of the unsaturated fatty acid profile	Roasting (hot air + infrared)	AFT	85–95%
Mahoney et al. 2020	Almonds	Natural	82–100 °C/2–3 min	Physical partitioning of the toxin into the water and removal of the perisperm	Risk of cross-contamination via process water	Blanching	AFT	76%
Morshedi and Razavi 2020	Pistachio	Natural	Infrared roasting under optimized conditions (70–90 V, 10 cm distance from the IR source)	Thermal degradation of aflatoxins promoted by infrared heating	Maintained acceptable quality attributes while reducing aflatoxins and microbial contamination	Roasting + infrared	AFT	90–95%
Valente et al. 2020	Hazelnut	Artificial	50 °C/20 h	Humidity control and spore inactivation of *A. flavus*	Temperatures above 50 °C increase the risk of lipid rancidity.	Drying + storage	AFT	100%
**Non-Thermal**								
Basaran et al. 2008	Pistachio/Hazelnut	Artificial	300 W/20 min (low pressure)	Bombardment by ions and reactive species (ROS/RNS)	Organoleptically acceptable; no microscopic changes	Cold plasma LPCP	AFT	50%
Basaran 2009	Hazelnut	Artificial	254 nm/9.99 J/cm²/6 h	Direct photodegradation and microbial DNA damage	Unpleasant (burnt) odors in shelled nuts	UV-C	AFT	25%
Jubeen et al. 2012	Pistachio	Natural/Artificial	265 nm/45 min	Chromophore absorption and cleavage of the terminal furan ring	Reduced efficacy in high-quality samples	UVC irradiation	AFT	92%
Siciliano et al. 2016	Hazelnut	Artificial	1000 W/12 min (N2 gas)	Direct attack on the C8–C9 bonds of the furan ring	Limited temperature rise (+ 28.9 °C); maintains quality.	Cold plasma DBD	AFT	70%
Hassanpour et al. 2021	Pistachio	Natural	Low-dose radiation/10 days	Prolonged exposure to natural gamma photons	Theoretical model for safe storage in silos	Gamma irradiation + MCNP simulation	AFB_1_	95%
Makari et al. 2021a	Pistachio	Artificial (spiked)	15 kV/20 kHz/180s	Oxidation by ROS/RNS and breakdown of molecular structure	Increase in MDA (lipid oxidation) and darker nuts	Gamma irradiation (Co-60)	AFB_1_	86.36%
Makari et al. 2021b	Pistachio	Artificial (spiked)	6 kGy (Cobalt-60)	Radiolysis of water and attack by free radicals (-OH)	Significant increase in MDA and oxidative stress	Cold plasma DBD	AFB_1_	52.42%
Esmaeili et al. 2023	Pistachio	Artificial	20 kV/15 min (Ar-Air 50%)	Ozonolysis and structural modification of the furan ring	Minimal impact on color, flavor, odor, and polyphenols.	Cold plasma DBD	AFT	65%
Zeraatpisheh et al. 2023	Pistachio	Artificial	80 W/15 min (simultaneous)	Synergism between photolysis and accelerated oxidation	Preservation of sensory and functional integrity (shelf-life)	UV-C + cold plasma	AFT	85%
Dinç et al. 2025	Mixed nuts	Natural	APCP, air gas, 600 V, 25 kHz, 2 bar, 2–16 min	ROS/RNS degrade aflatoxins by oxidation	Minor changes; moisture, FFA and peroxide affected, others stable	Atmospheric Pressure Cold Plasma	AFT	72.6%
**Combined methods**								
Sadeghi et al. 2022	Pistachio	Artificial	Roasting (120 °C for 20 and 40 min); Gamma irradiation (2.5 and 5 kGy); Microwave (1 and 2 min)	Physical degradation/inactivation of aflatoxins promoted by thermal treatment (roasting), microwave energy and gamma irradiation	No significant differences in sensory characteristics compared with the control samples.	Gamma irradiation/microwave	AFT	Confirmed reduction
Rastegar et al. 2017	Pistachio	Natural	Roasting (120–150 °C) with lemon juice and/or citric acid plus NaCl	Combined heat and acidic treatment degraded AFB_1_	High-temperature roasting may affect nutritional and organoleptic properties	Roasting + citric acid/lemon	AFB_1_	93.10%

AFT: Total aflatoxin; AFB_1_: Aflatoxin B_1_; AFB_2_: Aflatoxin B_2_

**Table 2:** Summary of the included studies using chemical methods on aflatoxin degradation

References	Tree nuts	Contamination Type	Processing Conditions	Mechanism	Quality Impact	Applied technique	Aflatoxin	Efficiency/Reduction
Akbas and Ozdemir 2006	Pistachio	Natural	9.0 mg/L/420 min	Oxidative attack on the C8–C9 double bond of the furan ring	Increase in peroxide value in ground walnuts; stable pH	Ozonization	AFT	24% (AFT)
Scussel et al. 2011	Brazil nut	Natural/Artificial	10.0 mg/L/90 min	Deep surface oxidation and sealing of the nut	MDA stability; improvement in sensory attributes	O3 reducing atmosphere	AFT	100% (AFT)
Jubeen et al. 2020	Mixed tree nuts	Natural	9% concentration/15 min	Acid hydrolysis of the lactone ring, forming the AFD_1_ derivative.	Conversion of AFB_1_ into a byproduct 450 times more mutagenic	Organic acids	AFT	99% (Walnut)/99.9% (Pistachio)
Ribeiro et al. 2020	Brazil nut	Natural/Artificial	SH (250 mg/L)/PAA (80 mg/L)	Inactivation of fungal conidia (sanitizing action)	Ineffective against pre-formed toxins; sensory acceptance maintained.	Hypochlorite/peracetic	AFT	Not effective
Atakan and Caner 2021	Hazelnut	Artificial	20 ppm/20 min	Electrophilic cleavage of the hypertoxic site of the furan ring	Severe 29% reduction in alpha-tocopherol; lipid oxidation	Ozonization	AFT	31.35% (AFT)/38.96% (AFB_1_)
Gammoh et al. 2023	Walnuts/Pistachio	Natural/Artificial	0.005% w/v/Drying 50 °C	Possible direct oxidative effect or enzymatic activation	Selenium fortification; without compromising sensory acceptance.	Selenium treatment	AFB_1_/AFG_1_	100% in walnuts
Kaminiaris et al. 2025	Pistachio	Natural	Lab and Field Trials	Inhibition of AF biosynthesis and control of A. flavus	High efficacy; requires regulatory validation of chemical residues.	Fungicides	AFT	100% (AFT)

AFT: Total aflatoxin; AFB_1_: Aflatoxin B_1_; AFB_2_: Aflatoxin B_2_

**Table 3:** Summary of the included studies applying biological methods on aflatoxin degradation

References	Tree nuts	Contamination Type	Processing Conditions	Mechanism	Quality Impact	Applied technique	Aflatoxin	Efficiency/Reduction
Rahaie et al. 2010	Pistachio	Artificial	12 h contact	Reversible physical adsorption to the yeast cell wall	Dependent on the initial concentration of the toxin	Immobilized Saccharomyces cerevisiae	AFB_1_	73% (AFB_1_)
Afsah-Hejri 2013	Pistachio	Artificial	Yeast biocontrol assay: 10⁷ cells/mL, 10 days at 28 °C	Biocontrol by saprophytic yeasts, reducing growth, sporulation, biomass, and AFB_1_ production.	Not reported	Saprophytic yeasts	AFB_1_	89.10%
Gorran et al. 2013	Pistachio	Artificial	Essential oils/extracts up to 4000 mg/L; 24 h/5 d	Inhibition of fungal growth, spore germination and AFB_1_	Not reported	Medicinal plants (Lamiaceae)	AFB_1_	97%
Doster et al. 2014	Pistachio	Natural (field)	Field application (wheat seeds)	Ecological competition and competitive exclusion of toxigenic strains	Pre-harvest management; no increase in rot incidence	Atoxigenic strain AF36	AFB_1_	20–45% (AFB_1_)
Ansari et al. 2015	Pistachio	Artificial	Optimized: 5 g kefir grains, 6 h, 30 °C	Binding/adsorption of aflatoxin by kefir microorganisms	Not reported	Kefir grains (RSM)	AFG_1_	96.80%
Hontanaya et al. 2015	Pistachio	Artificial	15 days/23 °C (storage simulation)	Gaseous release of allyl isothiocyanate (AITC); fungal enzymatic inhibition	Prolonged protection in active packaging; residual mustard odor	Mustard flours	AFT	87–100% (AFT)
Ansari et al. 2016	Pistachio	Artificial	KG 5–25%, 0–8 h, 20–60 °C; preheated at 70 °C	Binding/adsorption of aflatoxin by kefir grains	Not reported	Kefir grains	AFB_1_	96%
Farzaneh et al. 2016	Pistachio	Artificial	7 days/28 °C	Synergy between fengycin and surfactin (antifungal lipopeptides)	Reduction in the viability of Aspergillus flavus spores	Bacillus subtilis UTBSP1 (fengycin/surfactin)	AFB_1_	100% (AFB_1_)
Siahmoshteh et al. 2017	Pistachio	Artificial	8 days post-inoculation (dpi)	Enzymatic biodegradation via laccases and lipopeptide secretion	Spore inhibition with no reported sensory alteration	Bacillus subtilis/B. amyloliquefaciens	AFT	52.40% (AFT)/54.90% (AFB_1_)
Abdolshahi et al. 2018	Pistachio	Artificial	In vitro/Matrix contact	Selective chemical binding to the mannan component	Non-cellular alternative to prevent growth	Saccharomyces mannoprotein	AFB_1_	84.40% (AFB_1_)
Ben Taheur et al. 2019	Almonds	Artificial	7 days/25 °C	Production of organic acids (pH reduction) and bacteriocins	Fungal growth retardation and nutritional preservation	Lactic acid bacteria	AFB_1_/AFB_2_	85.27% (AFB_1_)/83.94% (AFB_2_)
Moradi et al. 2020	Pistachio	Natural	Infrared roasting under optimized conditions (70–90 V, 10 cm distance from the IR source)	Thermal degradation of aflatoxins by infrared heating, reducing fungal contamination and aflatoxin levels	No significant adverse effects on pistachio quality; acceptable physicochemical and sensory characteristics were maintained	Native yeasts	AFB_1_	98.30%
Pakizeh et al. 2022	Pistachio	Artificial (spiked)	15–30 days/25–46 °C	Interruption of AFs biosynthesis at the early stages	Probiotic benefits; sensory acceptance in nut pastes	Bifidobacterium lactis	AFB_1_	59.40% (AFB_1_)
Karami-Osboo et al. 2023	Pistachio	Artificial	9 days/28 °C	Release of phenolic compounds (thymol/carvacrol) via nanoparticles	Superior to free oil; long-lasting matrix protection	Encapsulated essential oil (Zataria multiflora)	AFT	99% (AFB_1_)

AFT: Total aflatoxin; AFB_1_: Aflatoxin B_1_; AFB_2_: Aflatoxin B_2_; AFG_1_: Aflatoxin G_1_

### Data analysis

The data extracted from the selected studies were summarized in Tables 1 and 2, and 3 and categorized by decontamination method. The studies were organized chronologically by year of publication, starting with the earliest study identified.

## Results and discussion

### Study selection

**Fig. 1:**
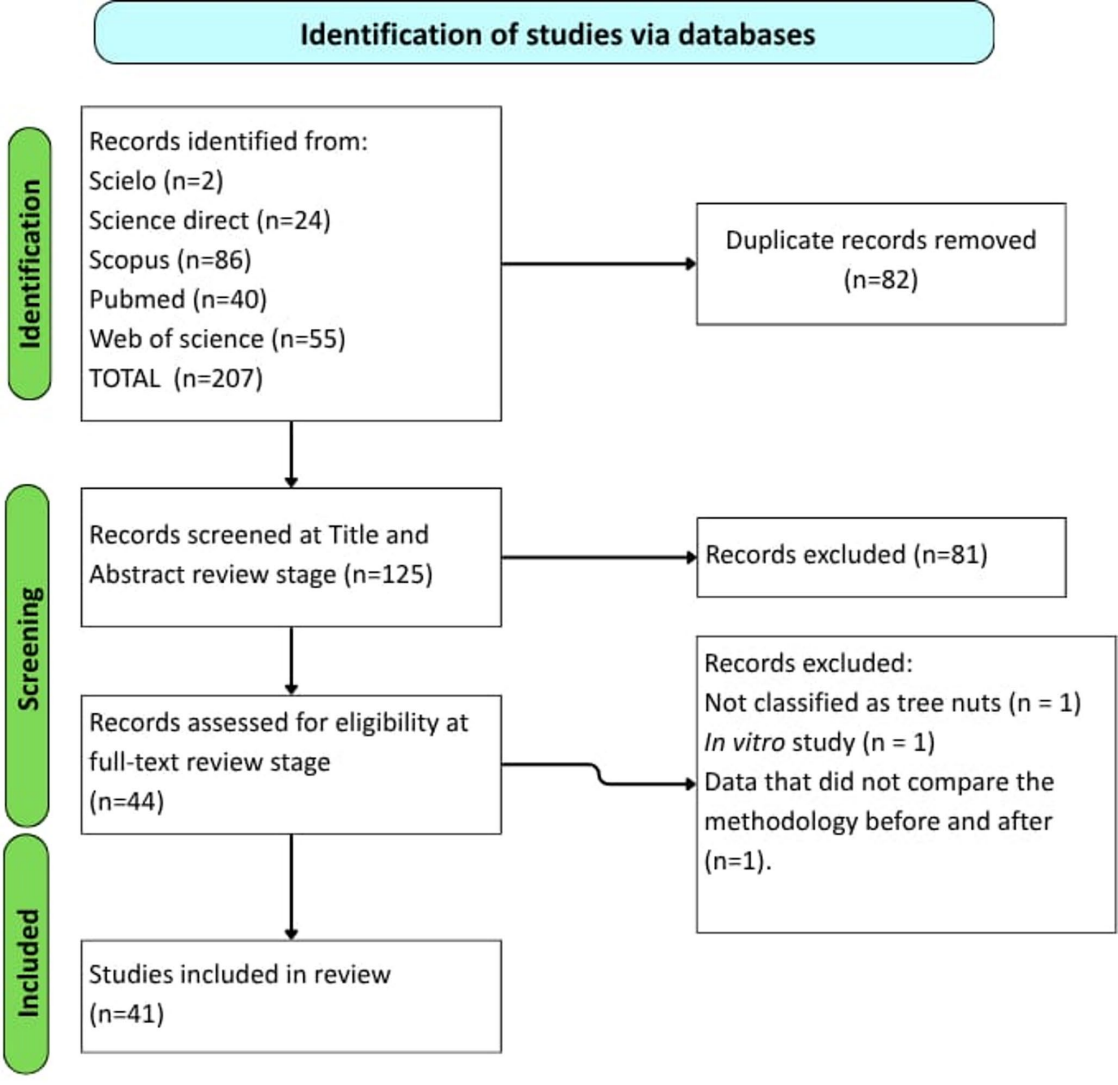
Preferred Reporting Items for Systematic Reviews and Meta-Analyses (PRISMA) flow diagram. Number of records identified at each stage of retrieval, screening, and data extraction

A total of 207 records were identified through database searches (PubMed, ScienceDirect, Scopus, and Web of Science). After 83 duplicates were removed, 124 unique records remained. Following abstract screening, 43 records were retained. Full-text assessment then resulted in the inclusion of 41 studies that met all eligibility criteria for this scoping review. Reasons for exclusion of the remaining studies are detailed in Fig. 1.

### Description of the included studies

The studies included in this scoping review examined various methods for decontaminating or reducing AFs in tree nuts, with most publications appearing between 2005 and 2025, reflecting increasing scientific interest in food safety and mycotoxin control. The most frequently assessed food matrices were almonds, pistachios, Brazil nuts, hazelnuts, and walnuts, although some studies included other varieties. The decontamination methods analyzed included physical treatments such as radiation, heat, and pressure; chemical methods using oxidizing agents and adsorbents; and biological approaches involving microorganisms or enzymes. Most studies focused on AFB_1_, recognized as the most toxic and prevalent AF, although some research included other variants such as AFB_2_, AFG_1_, and AFG_2_. Reduction rates varied widely, ranging from modest outcomes to efficiencies exceeding 80%, with effectiveness influenced by both the applied method and the specific characteristics of the tree nuts. The data extracted from the studies are summarized in Tables 1 and 2, and 3, organized by decontamination method and year of publication.

### Physical methods

Physical methods (Table 1) are interventions that act on environmental or food variables, such as temperature, humidity, radiation, pressure, or atmospheric composition, to interfere with mycotoxin stability. These methods do not involve the direct addition of chemical substances to the food, although they may trigger secondary chemical reactions, such as the thermal or oxidative degradation of the toxins. These treatments function primarily by removing or limiting factors essential to fungal metabolism, such as available water and oxygen, or by exposing the food to lethal or inhibitory conditions, such as heat or anoxic atmospheres. Thus, physical methods are notable for their applicability during post-harvest stages, as they leave no chemical residues and, in many cases, preserve the sensory and nutritional quality of the treated foods (Garcia-Cela et al. 2013).

### Thermal treatments: roasting, drying, and blanching

Roasting is an essential technological process that, in addition to enhancing sensory characteristics, reduces risks associated with mycotoxins. This treatment not only promotes the partial degradation of toxins but also lowers the moisture content of the pistachios, thereby limiting fungal growth and mycotoxin production during storage. Studies involving pistachio treatment at temperatures of 90, 120, and 150 °C for up to 90 min demonstrated that efficacy is dose-dependent: while treatment at 90 °C for 30 min resulted in modest reductions of 17% for AFB_1_ and 34% for total AFs, exposure to 150 °C for 120 min achieved reductions of up to 68% for AFB_1_ and 74% for total AFs (Yazdanpanah et al. 2005). However, extreme thermal conditions compromise the product’s physical qualities, imparting a burnt appearance that diminishes its commercial value.

The efficacy of heat treatment also varies depending on the processing method. Hot-air roasting proves superior to microwave roasting for degrading AFs in pistachios, achieving reductions of up to 93.1% for AFB_1_ and 90.8% for total AFs at 160 °C for 30 min, whereas microwave treatment (900 W for 5 min) reached maximum reduction rates of 47.8% and 43.4%, respectively (Siciliano et al. 2017; Morshedi and Razavi 2020). In other matrices, such as tree nuts, the combined application of forced drying and moderate roasting reduces moisture to levels below 4%, halting the metabolic activity of *Aspergillus flavus* and stabilizing the matrix against the synthesis of new toxins during storage (Costa et al. 2017). Blanching, though less commonly used for tree nuts due to moisture uptake, induces the thermal lysis of surface spores on almonds; however, its contribution to the degradation of already internalized AF molecules is limited by the high thermal stability of these chemical structures (Mahoney et al. 2020).

### Cold Plasma technology

Cold atmospheric pressure plasma is an emerging non-thermal technology that utilizes reactive oxygen and nitrogen species (ROS and RNS) to inactivate microorganisms and degrade mycotoxins. Plasma, considered the fourth state of matter, consists of ions, electrons, and highly reactive neutral species capable of breaking covalent bonds and initiating chemical reactions to inactivate microorganisms. In particular, the presence of the C8–C9 double bond in AFB_1_ and AFG_1_ renders these molecules more susceptible to oxidation, promoting furan ring opening and lactone modification, thereby reducing their toxicity (Siciliano et al. 2016).

Although the technique has demonstrated reductions of up to 83.7% for AFB_1_ in pistachios and approximately 70% in hazelnuts, industrial application faces challenges regarding the lipid stability of the matrix. Studies indicate that the treatment can induce oxidative stress, evidenced by a significant increase in levels of malondialdehyde (MDA), a byproduct of unsaturated fatty acid oxidation. Changes in MDA levels are closely linked to equipment operating parameters, such as voltage, frequency, and system pressure. The composition and density of the reactive species generated in the plasma are direct functions of electric field intensity and working gas pressure; increases in power supply voltage intensify free radical bombardment on the nut surface, which can accelerate lipid rancidity and compromise the product’s shelf life if conditions are not optimized. Thus, the technology’s success depends on the precise adjustment of these variables to maximize detoxification without exceeding the food’s oxidative tolerance threshold (Makari et al. 2021b; Esmaeili et al. 2023).

### Ultraviolet (UV-C) and gamma radiation

Electromagnetic radiation at different wavelengths offers distinct mitigation pathways based on photochemical and radiolytic effects. Irradiation with ultraviolet-C (UV-C) light, specifically targeting the 254 nm wavelength, demonstrated modest reductions of approximately 25% in surface AFs on hazelnuts; this limitation is attributed to the light’s low penetration power and the shadowing effect caused by shell roughness (Basaran 2009). In contrast, optimizing the spectrum to 265 nm for pistachios, almonds, and walnuts favorably altered degradation kinetics, achieving reductions of up to 100% for the AFG_2_ fraction; this wavelength coincides with the maximum absorption peak of the toxin’s conjugated ring system, thereby facilitating direct photolysis of the molecule (Jubeen et al. 2012).

Meanwhile, gamma radiation from Cobalt-60 sources operates via the radiolysis of residual water within the food, generating hydroxyl radicals (•OH) that break the chemical bonds of the AFs. Gamma irradiation of pistachios achieved up to a 5-log reduction in *A. flavus* population viability, eliminating the potential for recontamination, though it also induced a concomitant increase in malondialdehyde (MDA) levels within the fatty matrix (Makari et al. 2021a). Alternative strategies utilizing radioactive granite (RGR) beds achieved reductions of up to 95% in total AFs through prolonged exposure to low doses of natural radiation, thereby minimizing thermal damage to the matrix (Hassanpour et al. 2021). Methodological advances indicate that the development of hybrid Technologies, such as the synergistic effect between UV-C radiation and cold plasma, enables reductions of over 90% in AFs with shorter exposure times, thereby mitigating the individual deleterious impacts on the lipid and nutritional quality of walnuts (Zeraatpisheh et al. 2023).

### Technical challenges, scale-up constraints, and industrial readiness of physical methods

The transition of physical decontamination methods from experimental settings to industrial applications reveals a complex interplay of physical, economic, and regulatory bottlenecks. Traditional processing technologies, such as roasting, benefit from established operating costs and widespread machinery (TRL 8–9). However, when optimized to maximize toxin elimination, these conventional thermal methods cause irreversible sensory changes, particularly compromising the color and flavor of tree nuts (Yazdanpanah et al. 2005; García-Cela et al. 2013; Costa et al. 2017). Conversely, emerging non-thermal alternatives—including cold plasma and UV-C radiation—excel at preserving fatty acid profiles and primary organoleptic properties. Yet, their industrial adoption is severely constrained by a physical penetration barrier that limits their efficacy strictly to surface-level contamination (Ansari et al. 2016; Zeraatpisheh et al. 2023).

Scaling these non-thermal systems to a commercial level is further hindered by substantial initial capital investments and a lack of continuous-flow reactors capable of processing industrial volumes (in the range of tons per hour) while ensuring uniform exposure. Consequently, these systems remain largely positioned at lower technology readiness levels (TRL 4–6) for tree nut processing specifically. This technological immaturity is mirrored by regulatory uncertainty; no jurisdiction currently provides a dedicated regulatory pathway or validated critical limits for an aflatoxin-reduction claim based on cold plasma or UV-C. This regulatory void complicates their formal incorporation as Critical Control Points (CCP) within industrial HACCP plans (Ansari et al. 2016; Zeraatpisheh et al. 2023; Javed et al. 2025).

Even technologically mature alternatives like gamma irradiation (TRL 8–9) face distinct non-technical barriers, primarily driven by consumer wariness of irradiated foods and country-specific mandatory labeling or strict dose-ceiling requirements that restrict global market access (Makari et al. 2021a; Hassanpour et al. 2021). To bridge these operational gaps, the industry requires dedicated process-validation studies to establish reproducible critical limits (such as voltage, frequency, exposure time, and dose) tied to specific log-reduction targets for both *Aspergillus flavus* and AFB_1_ (Javed et al. 2025; Kumari et al. 2026). Until automated systems integrating optical sorting with targeted physical treatments become economically viable and standardized, physical decontamination will likely depend on hurdle technology frameworks. By combining moderate thermal interventions with modified atmospheres, processors can curb fungal proliferation and mitigate the impact of aflatoxins on the global nut supply chain without relying on a single, cost-prohibitive intervention (Siciliano 2016; Atakan and Caner 2021).

### Chemical methods

Chemical methods (Table 2) employ reactive agents to inactivate or degrade the molecular structure of AFs. Although they achieve the highest reduction rates, often approaching total elimination, their industrial application is contingent upon balancing the toxicological safety of by-products with the preservation of the nuts’ nutritional and sensory integrity.

### Oxidative degradation: ozonation and gaseous treatments

Ozonation stands out as a low-operating-cost technology that leaves no residues. Its mechanism relies on the strong oxidizing capacity of ozone (O_3_), which reacts with the double bonds in the AFs structure, specifically the C8–C9 bond of the furan ring in AFB_1_ and AFG_1_, yielding derivatives with lower molecular weight and reduced toxicity. Studies on hazelnuts and pistachios have demonstrated total aflatoxin (AFT) reductions ranging from 24% to 39%. However, in vacuum-packed Brazil nuts, ozone was the only method capable of degrading 100% of the AFs; in this application, it also reduced the nuts’ surface moisture and completely eliminated fungi and yeasts through its oxidative action on microbial cell wall and membrane components. Despite its efficacy, the treatment compromises nutritional quality; a reduction of up to 29% in α-tocopherol content was observed in hazelnuts, alongside an increase in peroxide values, indicating the onset of oxidative processes that could limit shelf life. For other nut varieties, product quality and sensory attributes were preserved (Akbas and Ozdemir 2006; Atakan and Caner 2021; Scussel et al. 2011).

### Structural modification: organic acids and acidification

The use of food-grade organic acids (citric, lactic, and propionic) emerges as a safe and effective alternative. The primary mechanism involves acid hydrolysis and the opening of the AFB_1_ lactone ring, converting it into a less toxic compound. Preliminary in vitro mutagenicity assays suggest that this byproduct may be up to 450 times less mutagenic than the original molecule (Jubeen et al. 2020). While this represents a promising safety advantage, these findings require further validation across different food matrices. Partial conversion of AFB_1_ to the less toxic AFB_2_ has also been observed, particularly following lactic acid treatment. Citric acid demonstrated reductions of up to 99% for AFB_1_ in walnuts and 93.1% in pistachios when combined with heat (120 °C). Although the treatment can reduce AFB_1_ toxicity, it is valued for preserving sensory qualities compared to aggressive oxidative methods (Jubeen et al. 2020; Rastegar et al. 2017).

### Emerging approaches: selenium and synthetic agents

Innovative decontamination strategies have demonstrated high potential for reducing AFs in tree nuts. For instance, a novel copper-based bio-MOF synthesized from non-toxic citric acid and glycine achieved over 95% aflatoxin removal in pistachio extracts within 10 minutes (He et al. 2025). The use of aqueous selenate solutions, followed by drying at 50 °C, resulted in the complete elimination of AFB_1_ in walnuts and pistachios without compromising sensory acceptance. Furthermore, by acting as an essential component of antioxidant enzymes, selenate contributed to reducing other mycotoxins, such as AFG_1_ and zearalenone, by up to 89%. In contrast, synthetic agents such as sodium hypochlorite, while effective at controlling fungi, showed limited ability to degrade toxins already present in Brazil nuts. Another method evaluated, the use of synthetic fungicides like cyprodinil and fludioxonil, demonstrated the capacity to eliminate up to 100% of AF production; however, it faces critical hurdles regarding consumer acceptance and strict regulatory restrictions due to the risk of chemical residues. Furthermore, the precise mechanisms by which these compounds interact with the toxins require further elucidation to ensure the safety and sustainability of commercial production systems (Kaminiaris et al. 2025; Gammoh et al. 2023; Ribeiro et al. 2020).

### Technical challenges, scale-up constraints, and industrial readiness of chemical methods

Decontamination efficiency is intrinsically linked to the potential for altering the food matrix, creating a delicate balance between safety and quality. Although highly effective at degrading surface toxins and controlling fungi, treatments such as ozonation and oxidative radiation pose the greatest risk of lipid oxidation and the loss of heat-sensitive nutrients, most notably $\:\alpha\:$-tocopherol. From an implementation standpoint, however, ozonation and organic-acid treatments are economically attractive because they rely on low-cost, readily available reagents. Ozone can be generated on-site from ambient air or oxygen, while food-grade citric, lactic, or propionic acids are easily sourced. These agents can often be retrofitted into existing washing, soaking, or drying lines without major capital investment, granting them comparatively high technology readiness levels (TRL 6–8) for industrial piloting (Akbas and Ozdemir 2006; Freitas-Silva et al. 2013; Atakan and Caner 2021; Jubeen et al. 2020; Rastegar et al. 2017).

Ozone additionally benefits from a Generally Recognized as Safe (GRAS) determination as an antimicrobial processing aid in several jurisdictions, which greatly facilitates its regulatory acceptance. Conversely, other chemical and biochemical alternatives remain at earlier development stages (TRL 3–5). For instance, selenium fortification is constrained by the need to establish safe upper intake limits for a micronutrient rather than a standard processing additive. Meanwhile, synthetic fungicides face steep consumer-acceptance barriers and strict residue-tolerance limits on ready-to-eat products (Gammoh et al. 2023; Kaminiaris et al. 2025).

The full industrial viability of any chemical agent depends on the transition to safe, residue-free treatments and the optimization of minimum effective doses that ensure toxicological safety without compromising the food’s functional and nutritional profile. Bridging this gap will require formal process characterization. Industrial validation must establish clear critical limits, monitoring frequencies, and corrective actions suitable for incorporating these treatments as validated Critical Control Points (CCP) within a HACCP plan. This technical framework must be supported by a comprehensive cost–benefit analysis that weighs reagent and equipment costs against reduced rejection rates and improved global market access (Mir et al. 2025).

### Toxicological safety of physical and chemical degradation by-products

A recurring limitation in the physical and chemical methods discussed above is that a reduction in aflatoxin concentration measured by HPLC or ELISA does not, by itself, confirm detoxification: the AFB_1_ or AFG_1_ molecule is chemically transformed rather than mineralized, and the resulting degradation products are not always fully characterized, either structurally or toxicologically. For ozone treatment, the opening of the furan ring at the C8–C9 double bond generates less mutagenic derivatives, the conversion of AFB_1_ to AFD_1_ mediated by citric or lactic acid is reported to reduce mutagenic potential by approximately 450-fold according to specific in vitro assays (Jubeen et al. 2020). However, it is critical to emphasize that these figures reflect initial screenings; comprehensive data on chronic toxicity, genotoxicity, and carcinogenicity for AFD1 remain scarce, particularly regarding its stability within complex tree nut matrices. For cold plasma, UV-C, and gamma irradiation, a recent comprehensive review of degradation mechanisms and cytotoxicity profiles concluded that while food irradiation up to the internationally accepted dose ceiling is considered safe overall, the detailed chemical identity and toxicological profile of many plasma and radiation-generated aflatoxin degradation products still need further elucidation before these technologies can be fully considered validated from a food security perspective (Kumari et al. 2026). Reactive oxygen and nitrogen species generated by cold plasma and gamma radiolysis also drive lipid oxidation, and the resulting oxidation by-products (aldehydes and other compounds associated with malondialdehyde) have toxicological and sensory implications for the treated matrix, which are rarely evaluated along with aflatoxin reduction data. Therefore, for all physical treatments of high-energy and strongly oxidative chemicals, industrial adoption should be conditioned on systematic toxicological characterization of the by-products of degradation, and not only on the percentage reduction of the main aflatoxin.

### Biological methods: sustainability and microbial interaction

Biological methods (Table 3) represent a promising frontier for food safety, utilizing live microorganisms, their enzymes, or secondary metabolites to inhibit fungal growth or permanently degrade AFs. Unlike aggressive chemical approaches, these strategies are valued for their inherent sustainability and Generally Recognized as Safe (GRAS) status. However, scaling up to an industrial level requires critical scrutiny, as nominal efficacy in in vitro assays is often limited by biological and structural bottlenecks within the food matrix (Ben Taheur et al. 2019).

### Microbial antagonism: probiotics, LAB, bacillus, and yeasts

The use of antagonistic microorganisms, predominantly lactic acid bacteria (LAB), probiotics, and yeasts, represents one of the most promising biocontrol strategies due to their GRAS status and their ability to preserve the sensory qualities of nuts. Decontamination in these matrices occurs via a dual pathway: active biopression, where metabolites inhibit fungal growth, and physical adsorption, where toxins are “sequestered” by the microbial cell wall.

LAB act primarily through the production of a wide range of antimicrobial compounds, including organic acids (lactic, phenyllactic, acetic), hydrogen peroxide, and bacteriocins. In almonds, the *Lactobacillus kefiri* strain FR7 demonstrated reductions of 85.27% for AFB_1_ and 83.94% for AFB_2_ after seven days of incubation. The inhibition mechanism is intrinsically linked to the acidification of the medium; studies show that neutralized supernatants (pH 7) lose their biocontrol efficacy, confirming that low pH disrupts the fungus’s metabolic activities and inactivates enzymes in the AF biosynthetic pathway. Furthermore, the application of *L. plantarum* supernatants resulted in the destruction of the cell wall and cytoplasmic membrane of *Aspergillus* spores, leading to the formation of membrane-bound vesicles and a loss of viability (Ben Taheur et al. 2019).

The application of probiotics extends to processed derivatives, such as pistachio paste, where the use of *Bifidobacterium lactis* achieved reductions of up to 73% in AFB_1_. Efficacy in these systems depends critically on the initial microbial load and storage time; significant reductions require specific CFU/g concentrations, which may alter the rheological properties and sensory profile of the final product. Other studies involving *L. rhamnosus* highlight the ability of these strains to reduce not only the toxin concentration but also its transport and metabolism in intestinal cells, thereby providing an additional layer of protection for the consumer (Pakizeh et al. 2022).

Kefir grains, which consist of a complex symbiotic association of lactic acid bacteria (LAB) and yeasts within a polysaccharide matrix, represent a highly efficient adsorption technology. Research on pistachios has demonstrated that kefir grains can remove up to 96% of AFB_1_ and 96.8% of AFG_1_ under optimized conditions (20 ng/g toxin, 10–20% grains, 6 h contact at 30 °C). Notably, prior heat treatment of the grains (70–110 °C) does not reduce their removal capacity, indicating that cell viability is not strictly necessary for adsorption; the process is driven by physical interactions between AF molecules and the structural components of the microbial cell wall (Ansari et al. 2015, 2016).

Yeasts, especially *Saccharomyces cerevisiae*, are widely recognized for their ability to bind AFs via cell wall β-glucans and mannans. In pistachios, immobilized *S. cerevisiae* reduced AFB_1_ levels by up to 73%, with efficiency increasing significantly following acid treatment of the cell wall, which exposes more binding sites. The kinetics of this process are extremely rapid, reaching saturation in just 2 to 3 h, an operational advantage over methods requiring long fermentation incubation periods (Abdolshahi et al. 2018; Rahaie et al. 2010).

Recent advances have led to the isolation of specific components to avoid unwanted microbial growth within the matrix. The use of purified mannoproteins, extracted from the outer layer of *S. cerevisiae*, achieved an 84.4% binding rate for AFB_1_. The mechanism suggests that AF molecules are adsorbed at mannose sites, altering the toxin’s structure into a form that is undetectable and less bioavailable (Abdolshahi et al. 2018; Pakizeh et al. 2022).

### Enzymatic biodegradation and biocontrol: *Bacillus* spp. and atoxigenic strains

Unlike passive adsorption, definitive biological detoxification relies on enzymatic biodegradation, wherein certain *Bacillus* bacteria secrete extracellular oxidative enzymes, such as laccases, to permanently cleave the AF lactone ring and eliminate their carcinogenic properties (Siahmoshteh et al. 2017). The *B. subtilis* strain UTBSP1 is notable for producing lipopeptides of the surfactin and fengycin families, which destabilize fungal membranes and reduce *Aspergillus flavus* spore viability by up to 94.1% (Farzaneh et al. 2016). In pre-harvest epidemiological management, competitive exclusion using native atoxigenic strains has established itself as a large-scale intervention tool. The deliberate introduction into the ecosystem of *A. flavus* lineages that have lost the ability to produce toxins, such as the *A. flavus* strain AF36, applied in commercial pistachio orchards at a rate of 11.2 kg/há, increases the frequency of the specific vegetative compatibility group to up to 93% of the total fungal population (Doster et al. 2014). This biological inundation displaces toxigenic populations from nutritional niches and reduces accumulated AFB_1_ levels at harvest by 20% to 45%, without increasing overall rates of fungal rot in commercial fruit.

### Natural bioactive compounds and active packaging

Bioactive compounds extracted from plant matrices represent another natural-based control strategy; essential oils from *Thymus daenensis* and *Satureja khozistanica* at a concentration of 375 mg/L completely suppress *A. flavus* mycelial growth and block AFB_1_ biosynthesis, while crude aqueous thyme extracts demonstrate direct degradative capacity, breaking down up to 97% of the free toxin at 2000 mg/L (Gorran et al. 2013).

Additionally, active packaging systems based on mustard flours serve as sources of glucosinolates that release allyl isothiocyanate (AITC) following enzymatic hydrolysis by myrosinase. In simulated closed storage atmospheres, the volatile action of AITC gas achieves reductions of up to 100% in AFT fractions in pistachios through the covalent cleavage of thiol and amino groups in essential fungal enzymes (Hontanaya et al. 2015).

However, the industrial application of free essential oils is limited by their high volatility and the risk of imparting strong residual odors that diminish the sensory quality of the nuts. To overcome this barrier, encapsulation technology using chitosan nanoparticles loaded with *Zataria multiflora* oil increases the AFB_1_ mitigation rate from 90.5% to 99.0% in actual pistachio matrices, providing controlled, sustained release of active phenols without compromising organoleptic attributes (Karami-Osboo et al. 2023).

### Technical challenges, scale-up constraints, and industrial readiness of biological methods

Although biological methods represent the most sustainable decontamination alternatives, their technological applicability and commercial scalability face significant hurdles. The primary physiological bottleneck lies in maintaining the viability and operational stability of these living agents within food matrices characterized by low water activity. In these dry environments, cellular survival drops sharply during storage, causing the robust efficacy demonstrated at the laboratory scale to diminish under real-world warehouse conditions due to drastic fluctuations in temperature and humidity (Pakizeh et al. 2022). Economically, while biological interventions benefit from strong consumer preference for “natural” and GRAS-labeled solutions and relatively low raw-reagent costs once a starter culture, enzyme, or bioactive extract is obtained, scaling production remains difficult. Producing stable, food-grade formulations, such as freeze-dried cultures, encapsulated enzymes, or purified mannoproteins, requires substantial investment in specialized fermentation and downstream-processing infrastructure that is not yet standardized for tree nut applications (Ben Taheur et al. 2019; Ansari et al. 2015, 2016).

These manufacturing constraints place most biological strategies at an early-to-intermediate technology readiness level (TRL 3–5), lagging behind the commercial maturity of thermal or ozone-based treatments. A notable exception is the use of atoxigenic-strain biocontrol, which is already commercially registered and applied at the field scale in some producing countries, achieving a high readiness level (TRL 7–8) (Doster et al. 2014). From a regulatory standpoint, acceptance is comparatively straightforward for GRAS microorganisms already established in fermented foods (such as *Lactobacillus*, *Bifidobacterium*, and *Saccharomyces cerevisiae*). However, novel enzymatic detoxifying agents and nanoencapsulated essential oils will likely require case-by-case safety and efficacy validation, including extensive migration and residue testing for the encapsulation materials (Karami-Osboo et al. 2023; Abdolshahi et al. 2018).

Overcoming these barriers and ensuring definitive food safety will depend on moving beyond theoretical generalizations. The future of commercial biological decontamination lies in the development of hurdle and barrier technologies that harmoniously combine sporulated or encapsulated living agents with moderate physical methods and smart packaging systems (Pakizeh et al. 2022).

### Pistachios: structural vulnerability and scientific dominance

In this review, the pistachio (*Pistacia vera L*.) has emerged as the primary experimental model for decontamination technologies. This prominence stems from the nut’s inherent susceptibility caused by “early split”, a phenomenon where the shell opens prematurely, affecting 80% to 94% of the fruit while still on the tree. This natural fissure, often exacerbated by damage from the navel orangeworm, serves as an entry point for *Aspergillus flavus* and *Aspergillus parasiticus* spores; their germination and subsequent AF biosynthesis are promoted by residual internal moisture (Rastegar et al. 2017). Economically vital for countries such as Iran, the USA, and Turkey, the fruit’s commercial value is constantly threatened by customs rejections. In the European market, pistachios account for the highest number of RASFF notifications. This situation is compounded by reports indicating up to a fivefold increase in median contamination levels over the last decade (Gharibzahedi and Savas 2025; Makari et al., 2021a). Although the porosity of the pistachio shell facilitates the penetration of gaseous and non-thermal agents (such as ozonation and cold plasma) to reach the kernel, extrapolating these results to other nuts is limited. Matrices like the Brazil nut possess a dense, sealed shell that acts as a formidable physical barrier against surface treatments, necessitating complex methodological adaptations, such as the use of vacuum, to ensure internal decontamination (Ansari et al. 2016; Scussel et al. 2017).

### Toward industrial implementation: process validation, HACCP integration, and cost–benefit considerations

Translating the reduction efficiencies reported in Tables 1, 2 and 3 into routine industrial practice requires more than demonstrating a favorable percentage reduction under laboratory or pilot conditions. Continuous-flow industrial processing of tree nuts occurs at scales of tons per hour, and any decontamination step considered for adoption must first undergo formal process validation, in which critical process parameters, equipment power or reagent dose, exposure or contact time, product moisture and load, and target log-reduction of both viable *Aspergillus* spp. and aflatoxin content, are established and shown to be reproducible across production batches, not only under the narrower conditions of a single spiked laboratory trial (Mateus et al. 2021; Mir et al. 2025). Once validated, a decontamination step with a demonstrated, reproducible critical limit can, in principle, be incorporated into a Hazard Analysis and Critical Control Points (HACCP) plan as a Critical Control Point, with defined monitoring procedures, corrective actions, and verification records; at present, however, only thermal treatments (roasting, drying) and, to a growing extent, ozonation have the process history and regulatory precedent needed to be integrated this way, whereas cold plasma, irradiation, and most biological interventions remain, for tree nuts specifically, in the process-development or pilot-validation stage (Table 4) (Javed et al. 2025; Ganesan et al. 2024).

**Table 4:** Comparative overview of aflatoxin decontamination methods in tree nuts: efficacy, quality impact, cost, industrial readiness, and regulatory status.

Method category	Representative technique(s)	Typical AF reduction (Tables 1, 2 and 3)	Nutritional/sensory impact	Relative cost*	Industrial scalability/TRL*	Regulatory/legal status	Key advantages	Key limitations	Key references
Thermal (roasting, drying, blanching)	Hot-air/infrared roasting; natural convection drying; blanching	17–100% (dose- and matrix-dependent)	Burnt appearance/sensory loss at high-efficacy settings; minimal impact at moderate settings	Low	High (TRL 8–9) – established industrial process	Fully accepted; conventional processing step, no special authorization	Low cost; widely available infrastructure; no chemical residues	Efficacy–quality trade-off; limited to matrices tolerant of heat	Yazdanpanah et al. 2005; García-Cela et al. 2013; Costa et al. 2017; Siciliano et al. 2017; Mahoney et al. 2020; Morshedi and Razavi 2020
Cold plasma	DBD, LPCP, APCP	50–86% (matrix- and parameter-dependent)	Minimal at optimized settings; risk of lipid oxidation (↑MDA) at high power/voltage	Moderate–High	Emerging (TRL 4–6) – pilot/lab scale for tree nuts	No dedicated framework for AF-reduction claims in most jurisdictions; generally residue-free	Non-thermal; preserves sensory quality at moderate doses; no residues	Surface-limited; oxidative by-products under-characterized; no industrial continuous-flow reactors yet	Siciliano et al. 2016; Makari et al. 2021b; Esmaeili et al. 2023; Dinç et al. 2025; Javed et al. 2025
UV-C/Gamma irradiation	254–265 nm UV-C; Co-60 gamma; RGR beds	25–100% (wavelength/dose- and matrix-dependent)	Variable: surface odor changes (UV-C); lipid oxidation (gamma, ↑MDA)	Moderate	Gamma: High (TRL 8–9)/UV-C: Moderate (TRL 5–7)	Country-specific labeling/authorization requirements; consumer-acceptance barrier	Strong microbial inactivation; established dose-safety ceiling	Consumer perception; degradation-product toxicology incomplete	Basaran 2009; Jubeen et al. 2012; Hassanpour et al. 2021; Makari et al. 2021a; Zeraatpisheh et al. 2023; Kumari et al. 2026
Ozonation	Aqueous/gaseous O3	24–100% (up to 100% in vacuum-packed Brazil nuts)	Risk of α-tocopherol loss (up to 29%) and ↑peroxide value	Low	High (TRL 6–8) – retrofit into washing/drying lines	GRAS antimicrobial status in several jurisdictions	No residues; low cost; dual fungicidal/detoxifying action	Lipid-oxidation risk; degradation by-products not fully characterized	Akbas and Ozdemir 2006; Scussel et al. 2017; Atakan and Caner 2021; Freitas-Silva et al. 2013
Organic acids	Citric, lactic, propionic acid ± heat	Up to 99% (AFB_1_)	Better sensory preservation than oxidative methods	Low	High (TRL 6–8)	Generally permitted as food-grade acidulants	Safe, effective, compatible with roasting	AFD_1_ by-product toxicology data still limited	Jubeen et al. 2020; Rastegar et al. 2017
Selenium/synthetic agents	Aqueous selenate; cyprodinil, fludioxonil	Up to 100% (selenium); up to 100% AF-production elimination (fungicides)	Selenium: no sensory loss, adds nutritional value; Fungicides: residue risk	Low–Moderate	Selenium: TRL 3–5; Fungicides: TRL 6–8 (regulatory barrier)	Selenium fortification limits not standardized; fungicide residues strictly regulated	High efficacy	Consumer acceptance (fungicides); fortification limits (selenium)	Gammoh et al. 2023; Kaminiaris et al. 2025; Ribeiro et al. 2020
Microbial antagonism (LAB, probiotics, yeasts, kefir)	L. kefiri, B. lactis, S. cerevisiae, kefir grains	73–96.8%	Generally preserves/enhances sensory and nutritional profile (GRAS)	Low–Moderate	Early–Intermediate (TRL 3–5)	GRAS status for established strains	Sustainable; dual bio-suppression/adsorption mechanism	Viability loss in low-water-activity matrices; scale-up variability	Ben Taheur et al. 2019; Pakizeh et al. 2022; Ansari et al. 2015; Ansari et al. 2016; Rahaie et al. 2010; Abdolshahi et al. 2018; Moradi et al. 2020
Enzymatic biodegradation/atoxigenic strains	Bacillus laccases/lipopeptides; A. flavus AF36	20–100% (lab, enzymatic); 20–45% AFB_1_ at harvest (field, atoxigenic)	Preserves matrix; atoxigenic strains applied pre-harvest, no product contact	Low–Moderate	Atoxigenic strains: TRL 7–8; Purified enzymes: TRL 3–4	Atoxigenic strains commercially registered in some countries (e.g., AF36, USA)	Permanent detoxification (enzymatic); displaces toxigenic populations (atoxigenic)	Enzyme production costs; geographic/regulatory restriction of strain release	Farzaneh et al. 2016; Siahmoshteh et al. 2017; Doster et al. 2014
Bioactive compounds/active-intelligent packaging	AITC-releasing mustard flour; encapsulated essential oils	87–100% (AITC); up to 99% (encapsulated EO)	Encapsulation preserves sensory quality; free EO risks residual odor	Low–Moderate	Early–Intermediate (TRL 3–5)	No dedicated framework for AF-reduction packaging claims	Passive, continuous protection during storage	Volatility of free EOs; packaging-migration/regulatory approval needed	Hontanaya et al. 2015; Gorran et al. 2013; Karami-Osboo et al. 2023

Cost–benefit considerations further differentiate these technologies. Thermal and ozone-based methods rely on comparatively low capital and operating costs and can frequently be retrofitted into existing roasting, washing, or drying lines, which explains their higher technology readiness levels (García-Cela et al. 2013; Freitas-Silva et al. 2013). Cold plasma, UV-C, and gamma irradiation require dedicated equipment and, for irradiation, access to a licensed facility, entailing higher upfront investment that is easier to justify for high-value, high-rejection-risk commodities such as pistachios than for lower-margin nuts (Makari et al. 2021a; Gharibzahedi and Savas 2025). Biological interventions, while inexpensive in terms of raw reagents, require investment in fermentation, encapsulation, or extraction infrastructure to produce stable, food-grade formulations at scale, and their cost-effectiveness will depend on whether they can be positioned as a value-added, “clean-label” alternative that commands a price premium sufficient to offset production costs (Pakizeh et al. 2022; Karami-Osboo et al. 2023). In every case, regulatory approval, including compliance with maximum residue limits, irradiation labeling requirements, and, for microbial or enzymatic agents, novel-food or biocontrol-agent registration, will determine market access as much as raw detoxification efficiency (Kaminiaris et al. 2025; Doster et al. 2014). Future research addressing this scoping review’s identified gap should therefore prioritize formal techno-economic assessments and continuous-flow pilot validations, particularly for cold plasma, encapsulated bioactive compounds, and atoxigenic strain applications, which currently show strong laboratory-scale performance but limited documented industrial deployment specifically in tree nuts (Javed et al. 2025; Mir et al. 2025).

Table 4 synthesizes, for each method category, the qualitative estimates of relative cost, industrial scalability/technology readiness level, and regulatory status discussed throughout this section, alongside the reduction efficiencies and quality trade-offs already summarized in Tables 1, 2 and 3.

### Future perspectives: toward digital and integrated aflatoxin management

Looking beyond the decontamination methods synthesized above, the future of aflatoxin management in tree nuts is likely to be shaped as much by digital and predictive technologies as by new detoxification chemistries. Machine learning (ML) and artificial intelligence (AI) are increasingly applied to two complementary tasks: predictive modelling of fungal growth and aflatoxin biosynthesis risk at the pre- and post-harvest stages, using climatic, agronomic, and storage variables, and rapid, non-destructive detection of contaminated kernels or batches, with reported classification accuracies exceeding 90% for several near-infrared- and imaging-based approaches (Focker et al. 2025; Deshmukh et al. 2025). Hyperspectral imaging, in particular, combined with deep-learning classifiers such as three-dimensional Inception–ResNet or attention-guided architectures, has shown the capacity to flag aflatoxin B_1_-contaminated almonds at the single-kernel level (Kabir et al. 2025), providing the technical basis for automated optical sorting systems capable of physically removing the most heavily contaminated nuts from a production line before, or in place of, chemical or physical detoxification.

Complementary developments include low-cost biosensors for on-site or in-line aflatoxin screening, such as recyclable Zr-MOF-engineered cotton fibers capable of simultaneous sensing and removal of toxins in liquid samples (He et al. 2023), intelligent packaging that couples active detoxification (e.g., the allyl isothiocyanate-releasing mustard-flour sachets discussed above) with colorimetric or electronic contamination indicators, and digital food-monitoring and traceability systems that log temperature, humidity, and processing parameters across the supply chain to support both regulatory compliance and rapid recall management (Gharibzahedi and Savas 2025; Hontanaya et al. 2015). None of these tools, on their own, degrades or removes aflatoxin from an already-contaminated kernel; their value lies in enabling real-time, risk-based decision-making, so that physical, chemical, and biological decontamination methods are applied selectively, to the batches or kernels that need them, rather than uniformly across an entire lot (Deshmukh et al. 2025).

The convergence of these digital tools with the decontamination methods reviewed here supports the broader concept of “hurdle technology”: multi-stage, synergistic interventions that combine, for example, AI-guided optical sorting to remove the most contaminated kernels, a moderate-intensity physical or chemical treatment (e.g., short-duration ozonation or cold plasma) calibrated to the reduced residual contamination load, and a biological or intelligent-packaging step to prevent recontamination during storage and distribution (Zeraatpisheh et al. 2023; Karami-Osboo et al. 2023). Realizing this integrated model at an industrial scale will require the technology-readiness gaps identified in Table 4 and in the preceding section to be closed through dedicated pilot- and industrial-scale validation, predictive models specific to tree nut species and growing regions, and harmonized international regulatory frameworks for both irradiation/plasma-treated and AI-sorted products (Focker et al. 2025; Kabir et al. 2025).

## Conclusion

Analysis of the 41 selected studies demonstrates that there is no single universal solution for AF decontamination in tree nuts. Instead, the viability of each intervention is determined by a rigorous assessment in which reduction efficacy must be balanced against the sensory and nutritional integrity of the nut matrix. A comparative synthesis of these trade-offs, together with qualitative estimates of relative cost, technology readiness, and regulatory status, is presented in Table 4. Physical methods, such as cold plasma and UV-C radiation, stand out for preserving initial organoleptic qualities but face penetration limitations that restrict their action to the nut’s surface; furthermore, they induce oxidative stress, as evidenced by increased malondialdehyde levels in pistachios. Among chemical approaches, ozonation and organic acids achieve reductions exceeding 90%, approaching total elimination. However, the loss of up to 29% of α-tocopherol in ozonated hazelnuts represents a significant nutritional trade-off that must be mitigated through dosage optimization. Biological methods, while sustainable and safe (GRAS), still lack industrial scalability due to the low stability of the living agents in low-water-activity matrices.

The prevalence of pistachios as an experimental model is justified by their intrinsic biological vulnerability (the “early split” phenomenon) and their significant economic weight regarding RASFF alerts. However, this review identifies a critical gap: the need to validate these technologies on nuts with closed shells, such as Brazil nuts, where the diffusion of decontaminating agents is physically hindered. The future of AF decontamination in nuts lies in the integration of multiple strategies that combine efficiency, toxicological safety, and economic viability. Furthermore, it will be essential to validate these techniques at an industrial scale, assess consumer acceptance, and meet international regulatory requirements to ensure safe and sustainable application within global production chains. Realizing this integration will increasingly depend on digital tools such as AI-assisted predictive modelling, hyperspectral optical sorting, and intelligent packaging, discussed in the Future Perspectives section above, working in concert with the physical, chemical, and biological methods reviewed here.

## Data Availability

No datasets were generated or analysed during the current study.

## Declarations

### AI Declaration

This study was financed by Carlos Chagas Filho Research Support Foundation of the State of Rio de Janeiro (FAPERJ E-26/200.270/2026); Coordination for the Improvement of Higher Education Personnel (CAPES − 0001); and the National Council for Scientific and Technological Development (CNPq − 309528.2025-9).
